# Enrichment of colorectal cancer associations in functional regions: Insight for using epigenomics data in the analysis of whole genome sequence-imputed GWAS data

**DOI:** 10.1371/journal.pone.0186518

**Published:** 2017-11-21

**Authors:** Stephanie A. Bien, Paul L. Auer, Tabitha A. Harrison, Conghui Qu, Charles M. Connolly, Peyton G. Greenside, Sai Chen, Sonja I. Berndt, Stéphane Bézieau, Hyun M. Kang, Jeroen Huyghe, Hermann Brenner, Graham Casey, Andrew T. Chan, John L. Hopper, Barbara L. Banbury, Jenny Chang-Claude, Stephen J. Chanock, Robert W. Haile, Michael Hoffmeister, Christian Fuchsberger, Mark A. Jenkins, Suzanne M. Leal, Mathieu Lemire, Polly A. Newcomb, Steven Gallinger, John D. Potter, Robert E. Schoen, Martha L. Slattery, Joshua D. Smith, Loic Le Marchand, Emily White, Brent W. Zanke, Goncalo R. Abeçasis, Christopher S. Carlson, Ulrike Peters, Deborah A. Nickerson, Anshul Kundaje, Li Hsu, on behalf of GECCO and CCFR

**Affiliations:** 1 Division of Public Health Sciences, Fred Hutchinson Cancer Research Center, Seattle, Washington, United States of America; 2 Joseph J. Zilber School of Public Health, University of Wisconsin-Milwaukee, Milwaukee, Wisconsin, United States of America; 3 Biomedical Informatics Program, Stanford University, Stanford, California, United States of America; 4 Department of Biostatistics, University of Michigan, Ann Arbor, Michigan, United States of America; 5 Division of Cancer Epidemiology and Genetics, National Cancer Institute, National Institutes of Health, Bethesda, Maryland, United States of America; 6 Service de Génétique Médicale, Centre Hospitalier Universitaire de Nantes, Nantes, France; 7 Division of Clinical Epidemiology and Aging Research, German Cancer Research Center (DKFZ), Heidelberg, Germany; 8 Division of Preventive Oncology, German Cancer Research Center (DKFZ) and National Center for Tumor Diseases (NCT), Heidelberg, Germany; 9 German Cancer Consortium (DKTK), German Cancer Research Center (DKFZ), Heidelberg, Germany; 10 Center for Public Health Genomics, Department of Public Health Sciences, University of Virginia School of Medicine, Charlottesville, Virginia, United States of America; 11 Division of Gastroenterology, Massachusetts General Hospital and Harvard Medical School, Boston, Massachusetts, United States of America; 12 Channing Division of Network Medicine, Brigham and Women's Hospital and Harvard Medical School, Boston, Massachusetts, United States of America; 13 Centre for Epidemiology and Biostatistics, Melbourne School of Population Health, The University of Melbourne, Melbourne, Victoria, Australia; 14 Division of Cancer Epidemiology C020, German Cancer Research Center (DKFZ), Heidelberg, Germany; 15 University Cancer Center Hamburg (UCCH), University Medical Center Hamburg-Eppendorf, Hamburg, Germany; 16 Division of Medical Oncology, Stanford School of Medicine, Stanford, California, United States of America; 17 Department of Molecular and Human Genetics, Baylor College of Medicine Center for Statistical Genetics, Houston, Texas, United States of America; 18 Ontario Institute for Cancer Research, MaRS Centre, South Tower, Toronto, Ontario, Canada; 19 Prevention and Cancer Control, Cancer Care Ontario, Toronto, Ontario, Canada; 20 Department of Medicine and Epidemiology, University of Pittsburgh Medical Center, Pittsburgh, Pennsylvania, United States of America; 21 Department of Internal Medicine, University of Utah Health Sciences Center, Salt Lake City, Utah, United States of America; 22 Department Genome Sciences, University of Washington, Seattle, Washington, United States of America; 23 University of Hawai’i Cancer Center, Honolulu, Hawai’i, United States of America; 24 Department of Epidemiology, University of Washington, Seattle, Washington, United States of America; 25 Division of Hematology, University of Ottawa, Ottawa, Ontario, Canada; 26 Clinical Epidemiology Program, Ottawa Hospital Research Institute, Ottawa, Ontario, Canada; 27 Department of Genetics, Stanford University, Stanford, California, United States of America; 28 Department of Biostatistics, University of Washington, Seattle, Washington, United States of America; University of Texas Health Science Center at Houston, UNITED STATES

## Abstract

**Background:**

The evaluation of less frequent genetic variants and their effect on complex disease pose new challenges for genomic research. To investigate whether epigenetic data can be used to inform aggregate rare-variant association methods (RVAM), we assessed whether variants more significantly associated with colorectal cancer (CRC) were preferentially located in non-coding regulatory regions, and whether enrichment was specific to colorectal tissues.

**Methods:**

Active regulatory elements (ARE) were mapped using data from 127 tissues and cell-types from NIH Roadmap Epigenomics and Encyclopedia of DNA Elements (ENCODE) projects. We investigated whether CRC association p-values were more significant for common variants inside versus outside AREs, or 2) inside colorectal (CR) AREs versus AREs of other tissues and cell-types. We employed an integrative epigenomic RVAM for variants with allele frequency <1%. Gene sets were defined as ARE variants within 200 kilobases of a transcription start site (TSS) using either CR ARE or ARE from non-digestive tissues. CRC-set association p-values were used to evaluate enrichment of less frequent variant associations in CR ARE versus non-digestive ARE.

**Results:**

ARE from 126/127 tissues and cell-types were significantly enriched for stronger CRC-variant associations. Strongest enrichment was observed for digestive tissues and immune cell types. CR-specific ARE were also enriched for stronger CRC-variant associations compared to ARE combined across non-digestive tissues (p-value = 9.6 × 10^−4^). Additionally, we found enrichment of stronger CRC association p-values for rare variant sets of CR ARE compared to non-digestive ARE (p-value = 0.029).

**Conclusions:**

Integrative epigenomic RVAM may enable discovery of less frequent variants associated with CRC, and ARE of digestive and immune tissues are most informative. Although distance-based aggregation of less frequent variants in CR ARE surrounding TSS showed modest enrichment, future association studies would likely benefit from joint analysis of transcriptomes and epigenomes to better link regulatory variation with target genes.

## Introduction

Colorectal Cancer (CRC) is a leading cause of cancer-related morbidity and mortality worldwide [1]. An understanding of the genetic etiology of CRC may inform therapeutic development and improve the effectiveness of targeted preventive strategies. To date, we know of several very rare genetic mutations that increase risk for hereditary syndromes predisposing to CRC [2]; overall these very rare high penetrance mutation account for less than 3–5% of the heritability. Recently, genome-wide association studies (GWAS) have discovered 48 independent common (minor allele frequency, MAF > 5%) autosomal genetic variants associated with CRC as well. While an estimated 12–35% of CRC risk is attributed to genetic factors [3,4] the additive variance from known common CRC variants account for 1–4% [4–7] of the narrow-sense heritability of CRC. Cumulatively, common genetic variation is estimated to explain approximately 7–8% of CRC heritability [5], suggesting that while larger CRC GWAS will discover additional common variant associations, a substantial fraction of CRC heritability may be explained by rare variants.

Although GWAS enable the discovery of common variant associations, array-based GWAS are not well-suited to evaluate the role of less frequent (0.1% < MAF < 1%) variants on CRC risk. Whole-genome sequencing (WGS) studies offer an alternative approach to array-based GWAS for investigating rare variants. Specifically, WGS can be performed on a reference set that is used to impute sequenced variants into samples with existing genome-wide genotype array data [6,7]. Recent studies have successfully applied this approach to identify novel low-frequency variants associated with complex traits [6,8,9]. Despite these successes, genotype imputation in large numbers of samples is still underpowered for detecting trait associations with less frequent genetic variants.

The power of rare-variant association methods (RVAM) can be improved in a variety of ways. By utilizing functional genomic data, tests of single variant trait associations can be restricted to regions of putative functional significance (or weighted accordingly). Power can also be improved by using aggregate testing for rare-variants. In aggregate RVAM, variants are aggregated within a set (typically a gene) and tested for association with a phenotype. To date, most RVAM have focused on the 1–2% of the genome that encodes for proteins [10,11] where reference gene annotations, such as RefSeq Genes [12], GENCODE [13], or the consensus coding sequence (CCDS) [14], provide functionally relevant units for analysis. To conduct a sufficiently powered *genome-wide* search for rare and less frequent variant associations, an important challenge is to identify units of analysis outside of coding regions that are biologically meaningful and enriched for associations with CRC.

**Fig 1:**
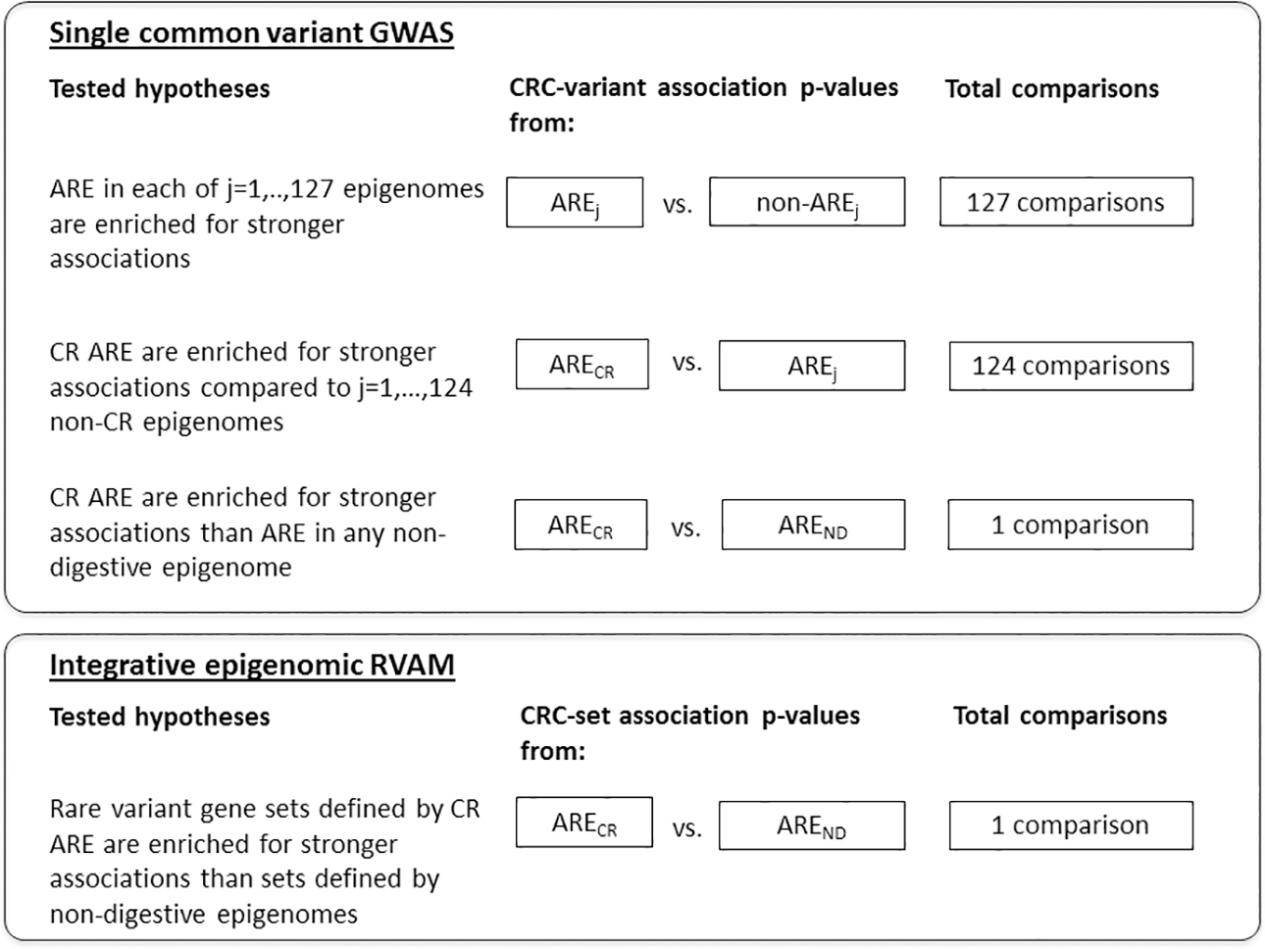
Analysis approaches for assessing ARE enrichment of stronger CRC associations with common and less frequent variants. ARE, Active Regulatory Elements; CR, Colorectal; ND, Non-digestive; Fig 1 describes the series of analyses performed examining the enrichment of ARE for more significant *common single* variant CRC associations from GWAS and *less frequent variant* set associations from an integrative epigenomic RVAM.

To define non-coding regulatory regions that modulate gene expression, we used functional genomic data from the NIH Roadmap Epigenome project and ENCODE in 127 different tissues and cell-types, including 3 colorectal epigenomes [15]. We then identified chromatin accessible genomic regions that overlapped nucleosome signals for enhancer and promoter states [15]. We refer to these regions as active regulatory elements (ARE) because they often demark regions of transcription factor binding that in turn modulate transcript abundance of target genes. Finally, we utilized enrichment-based methods to evaluate the extent to which ARE may be used to inform both single variant- and aggregate set-based analyses of rare and less frequent-variant associations. To do so, common single variant GWAS and integrative epigenomic RVAM analyses were performed in 12,661 CRC cases and 14,361 controls. A panel of 610 CRC cases and 309 controls with low-coverage WGS data was used to impute allelic dosages for variants that were not directly genotyped in the GWAS data. Tests of enrichment were conducted using the association results (p-values) from single-variant and aggregate RVAM (Fig 1).

## Results

### ARE enrichment of stronger CRC associations with common variants

**Fig 2:**
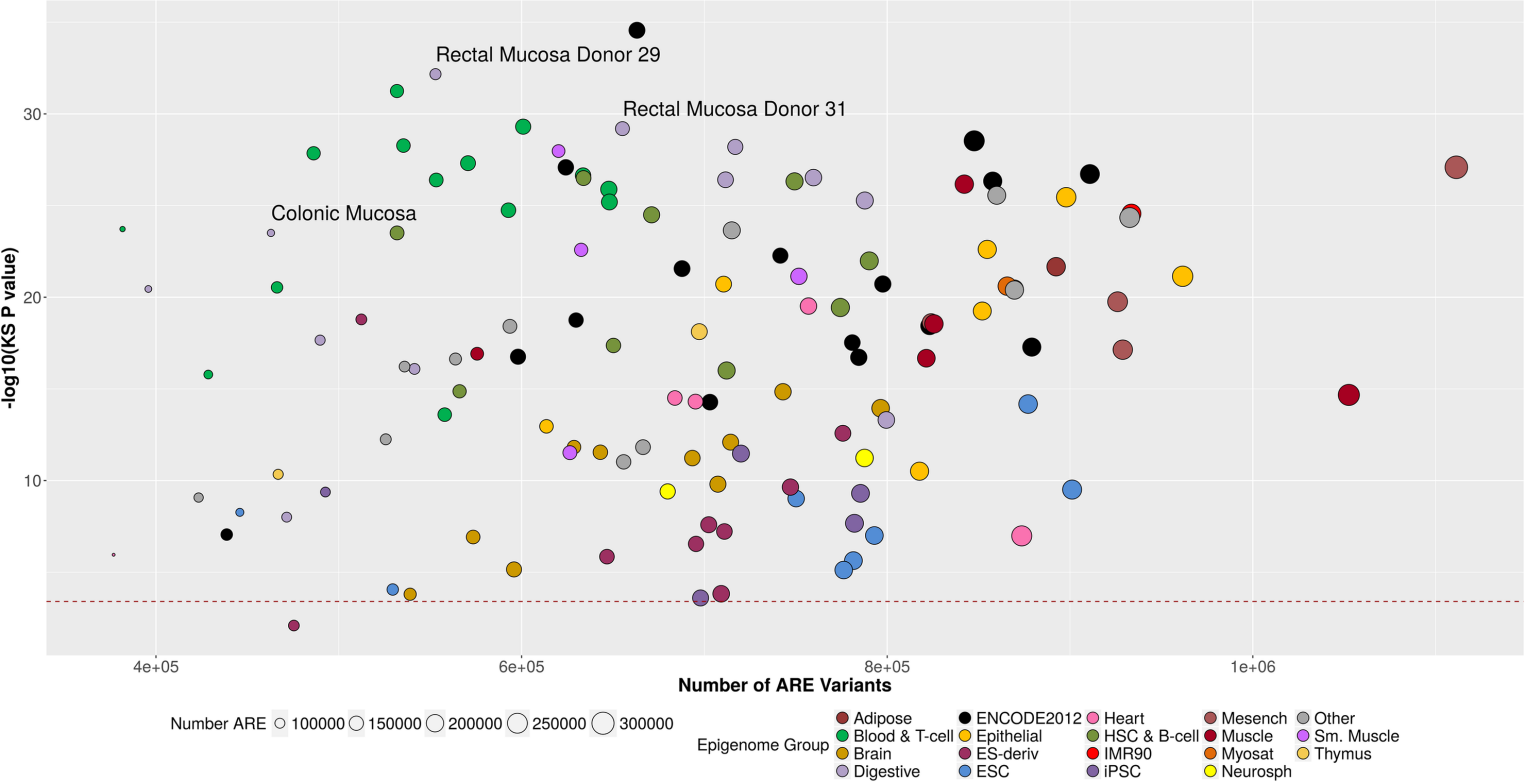
ARE enrichment of stronger GWAS CRC p-values. Each point in this scatter plot corresponds to one of the 127 tissues and cell-types examined for ARE enrichment of more significant single common variant association p-values with CRC. The x-axis shows the number of variants in ARE examined for the corresponding tissue or cell-type. The y-axis scale is the–log 10 × the p-value from the KS test comparing the distribution of CRC-variant association p-values for variants inside and outside of ARE. The size of the point corresponds to the number of ARE in the tissue or cell-type. The color of the point represents membership to an epigenomic group, based on a hierarchal clustering of the AREs. ARE of digestive tissues (in light purple) and immune cell types (in two shades of green) were more significantly enriched (lower KS test p-values). The range of ARE and ARE variants spreads across the high and low KS P-values, suggesting that the difference in number of ARE across tissues is not biasing the enrichment results. The red dash line corresponds to the Bonferroni p-value threshold correcting for 127 comparisons (0.05/127 = 4 × 10^−4^).

First, we tested whether ARE were enriched for common variant (MAF ≥ 1%) CRC associations (low p-values) when compared to non-ARE regions of the genome. On average there were 692,408 (ranging from 376,794 to 1,111,337) variants positioned in ARE of the 127 tissues and cell-types examined in this analysis. For comparison, there was an average of 7,585,498 (ranging from 7,901,112 to 7,166,569) common variants outside of the ARE regions. The number of ARE variants in a tissue was highly correlated with the number of ARE in a tissue (Pearson correlation r^2^ = 0.93). The KS test enrichment p-value, however, was not correlated with the number of ARE variants in a tissue or cell-type (Pearson correlation r^2^ = 0.02). For the 127 tissue and cell-type specific ARE, all but neuron cultured cells showed statistically significant (α = 0.05/127 comparisons = 3.9×10^−4^) enrichment of CRC associations from the one-sided Kolmogorov-Smirnov (KS) test (S1 Table). We found that ARE variants with low CRC association p-values were more significantly enriched (smaller KS p-values) in digestive tissues, including the 3 colorectal tissues, and immune cell types (Fig 2). Embryonic stem cells (ESC), induced pluripotent stem cells (iPSC), and brain cell-types had weaker ARE enrichment.

**Fig 3:**
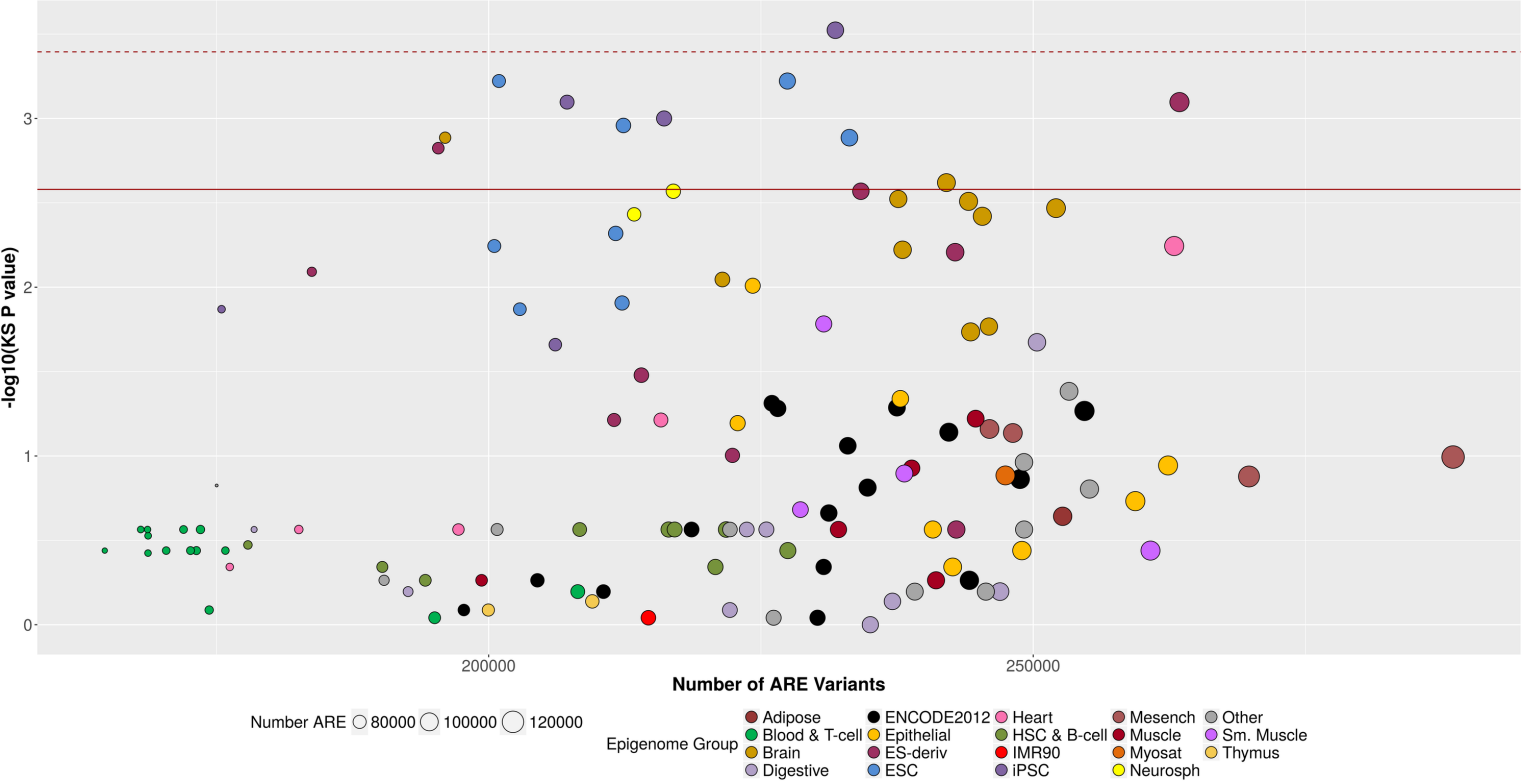
CR-specific ARE enrichment of stronger GWAS CRC p-values. Each point in this scatter plot corresponds to an enrichment result comparing CR ARE (n = 108,297) to ARE in one of the 124 non-colorectal tissues and cell-types tested. The x-axis shows the number of variants in ARE examined for the corresponding non-colorectal tissue or cell-type (n = 270,030 CR ARE variants). The y-axis is the–log 10 × the p-value from the KS test comparing the distribution of CRC association p-values for variants inside CR ARE versus those inside ARE of a non-colorectal tissue and cell-type. The size of the point corresponds to the number of ARE in the tissue or cell-type. The color of the point represents membership to an epigenomic group, based on a hierarchal clustering of the AREs. In comparison to ARE of digestive tissues (in light purple) and immune cell types (in two shades of green), CR-specific ARE did not exhibit additional enrichment. The strongest enrichment was observed for induced pluripotent stem cells (iPSCs), embryonic stem cells (ESC), and ESC-derived cells, and Brain cell-types. The red dash line corresponds to the Bonferroni p-value threshold correcting for 124 comparisons (0.05/124 = 4 × 10^−4^). The red solid line corresponds to the Bonferroni p-value threshold correcting for 19 epigenomic group comparisons (0.05/19 = 3 × 10^−3^).

Next, we investigated whether colorectal (CR) tissue-specific ARE were enriched for low p-values of CRC-variant association in comparison to the ARE of non-CR tissues and cell-types. To do so, we pooled ARE in the three reference CR tissues into a single set and made comparisons to ARE in each of the remaining 124 tissues and cell-types. We then generated an empirical p-value using adaptive resampling with up to 10,000 iterations. After adjustment for 124 multiple comparisons, CR ARE were only enriched for low p-values of CRC-variant association in comparison to ARE in iPSC cells (p = 3 × 10^−4^; S2 Table, Fig 3). However, previous cluster analysis [15] revealed 19 groups of similar epigenomic landscapes (defined as the Epigenomic Group in S2 Table) that correspond to biologically relevant groups (such as digestive tissues and embryonic stem cells, S1 Fig). After adjustment for 19 comparisons (corresponding to anatomical locations with similar epigenomes), CR ARE were enriched for low p-values of CRC-variant associations in comparison to ARE from induced pluripotent stem cells (iPSCs), embryonic stem cells (ESC), and ESC-derived cells (p-values ranging from 3 × 10^−4^ to 0.001). These epigenomes are derived from tissues and cell-types that are anatomically distant from CR tissues and with more divergent epigenomes (S1 Fig) [15]. Finally, when pooling the ARE of 115 non-digestive tissues and cell-types and 3 CR tissues we found significant enrichment of low p-values of CRC-variant associations in CR ARE versus non-digestive ARE (KS test p-value = 0.0035, empirical p-value = 1.0×10^−4^ based on 10,000 resampling iterations).

**Fig 4:**
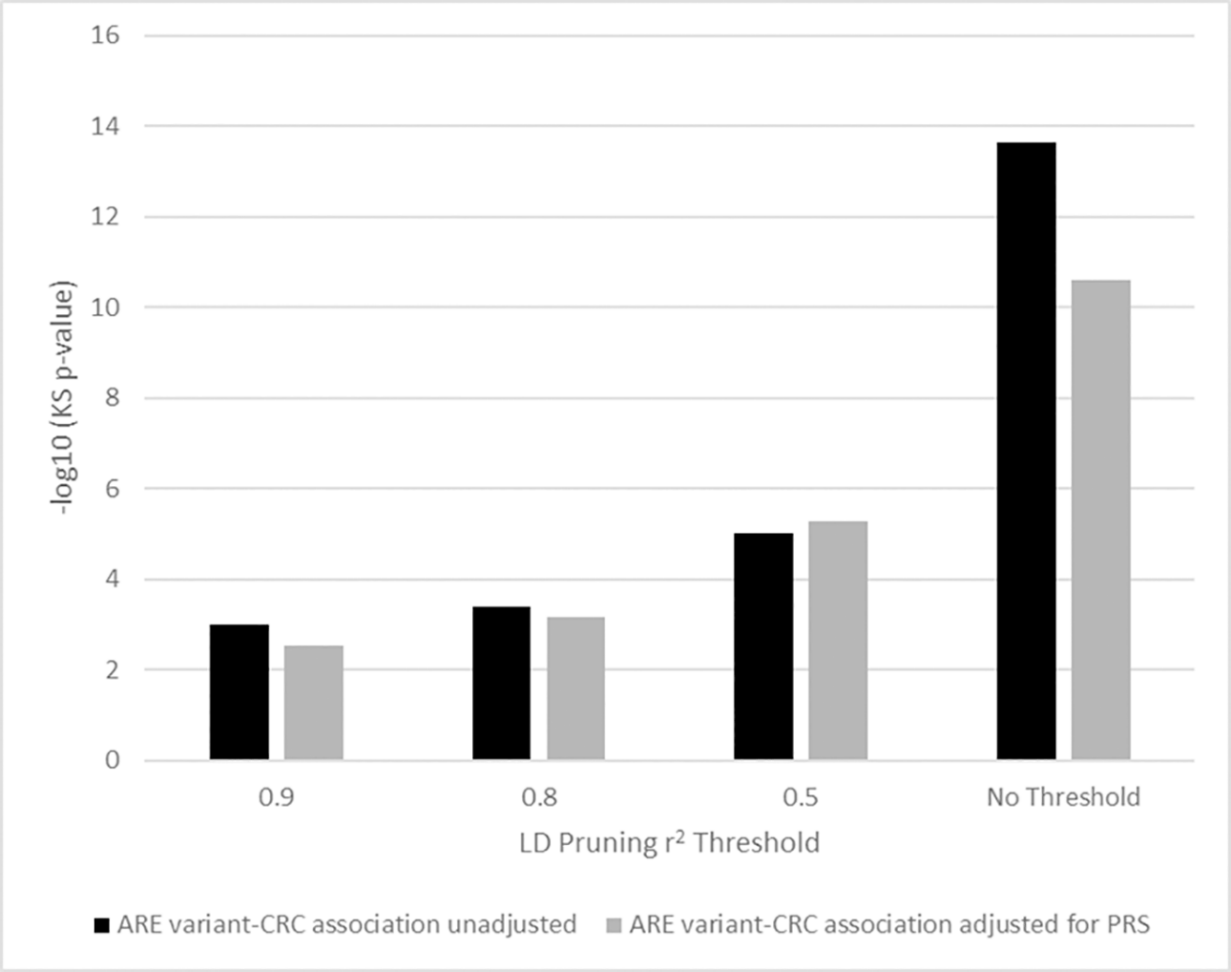
CR-specific ARE enrichment of stronger GWAS CRC p-values accounting for known loci and variant correlation. Variant-CRC association p-values for CR ARE variants were compared to ARE variants of all non-digestive tissues. The y-axis shows the–log10(KS test p-value) reflecting the significance of the enrichment. Enrichment analyses were performed for single variant-CRC association p-values from GWAS unadjusted for known loci (shown in black) and from GWAS that included a polygenic risk score (PRS) in the model (shown in gray). Results across different LD pruning schemes are shown using three different correlation r^2^ thresholds. For each threshold, LD blocks were defined as sets of correlated CR ARE variants or ARE variants from non-digestive tissues with r^2^ greater than or equal to 0.9, 0.8, or 0.5. For each LD block, a priority pruning scheme was employed selecting the ARE variant with the strongest CRC association p-value. Enrichment tests were repeated for each pruning threshold and compared to enrichment results without LD pruning (‘No Threshold’).

To investigate whether our results were sensitive to patterns of linkage disequilibrium (LD) near ARE, we employed a priority pruning scheme, where the variant with the lowest CRC association p-value in an LD block (defined at Pearson correlation r^2^ thresholds) was maintained for enrichment analysis. After LD pruning we still found significant enrichment of stronger CRC-variant associations in CR ARE in comparison to ARE combined across non-digestive tissues and cell-types at LD thresholds of r^2^ = 0.9, 0.8, and 0.5 (KS p-value = 9.6 × 10^−4^, 4.1 × 10^−4^, and 9.5 × 10^−6^, respectively; S4 Table and Fig 4). To further explore how much enrichment is driven by known CRC loci we repeated the single common variant GWAS adjusting for a polygenic risk score (PRS) based on the 48 known index variants and repeated the enrichment analysis using all variants and at the three LD pruning thresholds. At each of the pruning thresholds we observed similar enrichment for stronger CRC-variants association p-values in CR ARE compared to ARE of non-digestive tissues. In the absence of LD pruning the KS p-value was diminished after adjusting for PRS, but still highly significant (KS p-value = 2.5 × 10^−11^ versus 2.2 × 10^−14^, Fig 4).

### CR ARE enrichment of rare variant CRC associations from aggregate tests

**Fig 5:**
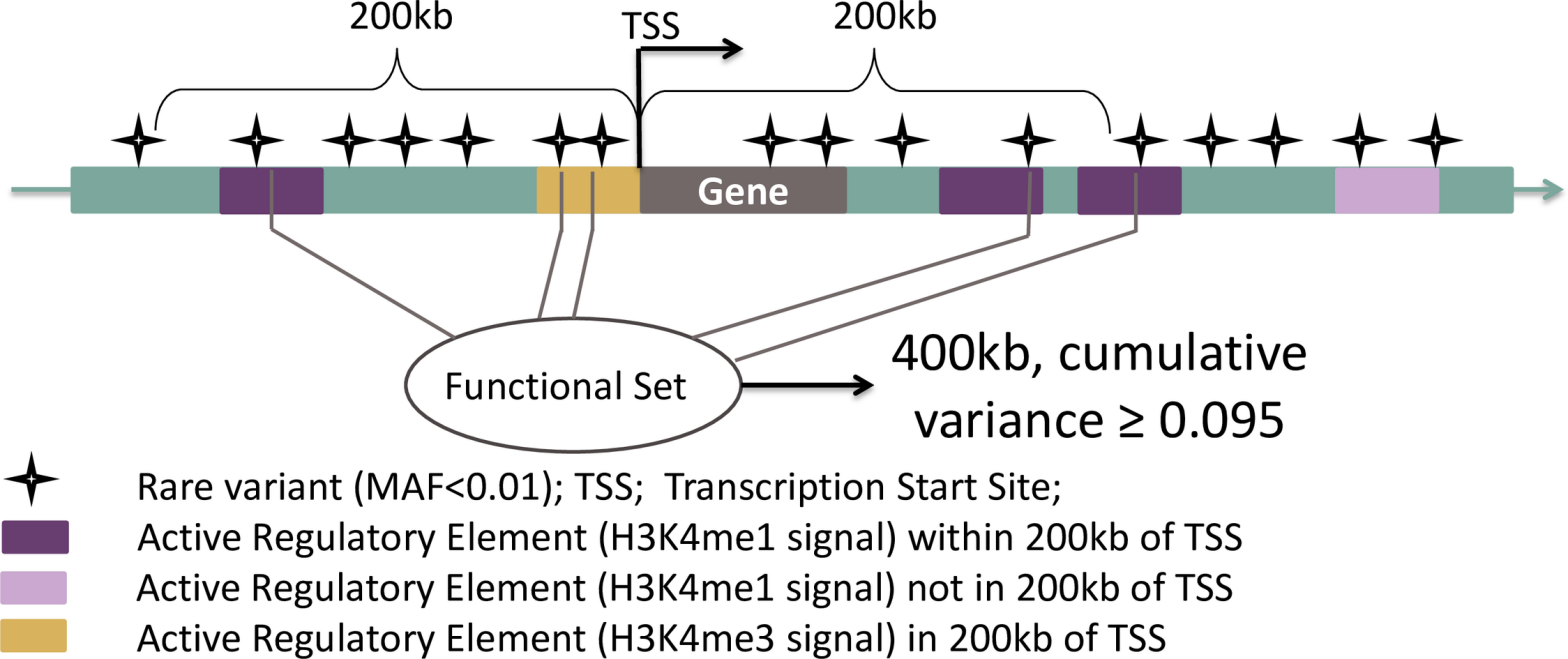
Rare variant test set. Variant sets were anchored on Transcription Start Sites (TSS) as defined by protein coding gene transcripts with validated RefSeq records. If a gene had multiple TSS, the 5'-most and 3'-most TSS were used as anchors. Accordingly, variants overlapping ARE within 200Kb of a TSS were pooled into a test set.

To explore whether CR ARE were enriched for low p-values for aggregate RVAM CRC associations, we leveraged evidence suggesting that regulatory elements are often located within 200kb of their target gene(s) [16]. Accordingly, we defined sets of rare variants (MAF < 1%) by pooling ARE within 200kb upstream or downstream of transcription start sites (TSS) from RefSeq [12], (Fig 5 and S3 Fig). We pooled CR-specific ARE as well as ARE in non-digestive tissues/cell-types and ran set-based association tests for CRC (see Methods) in both. We refer to this as an integrative epigenomic RVAM.CR-specific sets were enriched for rare-variant CRC-associations compared to sets specific to non-digestive tissues and cell-types (p-value = 0.029).

## Discussion

Consistent with the results from previous studies [17], we found that ARE (defined using the Roadmap and ENCODE epigenomic data) were enriched for variants associated with a complex human disease, in our case, CRC. Interestingly, when comparing CR ARE directly to ARE defined in other tissues and cell-types we found significant enrichment of low p-values in comparison to anatomically distant tissues and cell-types. We did not observe enrichment when comparing CR ARE to ARE defined in other digestive tissues. Contrary to our expectations, we also observed minimal enrichment in comparison to most of the other tissues and cell-types. This suggests that although presence of the ARE in CR tissue is important, there may be low specificity of the ARE harboring risk variants in CR tissue in comparison to ARE of other epigenomes, particularly those from similar anatomical locations and regulatory landscapes. That being said, we did observe stronger enrichment of low CRC-variant association p-values inside ARE of digestive and immune cell-types when compared to regions outside of ARE relative to the enrichment observed from other cell-types and tissues. This suggests that these epigenomes may be most relevant for honing in on putative functional variants. It is possible that in light of the limited sample sizes that are currently available for CR tissues (n = 3), the reference ARE maps are incomplete. If this is true, inclusion of all digestive tissues with similar epigenomic landscapes may help identify missed CR ARE that are shared across all digestive tissues.

In addition, for aggregate testing of rare-variant associations, we found that sets based on CR-specific ARE were enriched compared to sets based on ARE from non-digestive cell-types. We acknowledge that physical proximity of enhancers to genes has limited predictive accuracy for identifying the gene targets [18]. However, even though the rare-variant analysis was anchored on genes by considering ARE within 200kb of TSS, our method for defining units of analysis represents a significant advance over the exome-only analyses that are the current standard. Given that most of the common CRC loci identified thus far are positioned outside of the exome, our enrichment results suggest that rare variant associations will similarly implicate non-protein coding regulatory mechanisms. The lower enrichment observed for rare variant associations in comparison to common variants likely reflects both incomplete mapping of ARE and the employed pooling scheme. Future approaches could benefit from integration of epigenomic, transcriptomic, and genomic data in order to group ARE linked to regulation of the same target gene(s) through expression quantitative trait mapping.

Our findings have broad implications for future studies aimed at understanding the genetic etiology of CRC. First, we demonstrated that regulatory variation may play an important role in the inherited susceptibility to CRC. Specifically, using an LD r^2^ ≥ 0.5 threshold, we considered all variants that tag one of the 48 known, common-variant loci for CRC risk (S3 Table). We found that 36 of the 48 index variants (73%) tagged a variant located in a CR ARE. Of those not positioned within a CR ARE, 5 were in the AREs of stomach mucosa, duodenum mucosa or relevant immune cells. Second, by showing that CRC associations are enriched in ARE, we demonstrate that methods for discovering new associations that prioritize variants in ARE may gain statistical power over agnostic approaches. Finally, with rare-variant association studies expanding beyond the exome, there are as yet no gold-standard approaches for constructing non-coding units of analysis for rare-variant association testing. The ARE we defined represent a logical non-coding unit of analysis that permit aggregate rare-variant association tests such as Mixed Effects Score Test (MiST) [19], Sequence Kernel Association Tests (SKAT) [20], or Combined and Multivariate Collapsing Method (CMC) [21].

This study has a number of strengths. We utilized a large collection of WGS data to investigate rare, low-frequency, and common variants across the genome. We are also confident that the p-values for association with CRC risk are well-calibrated due to the large sample sizes in GECCO and CCFR (over 27,000 samples). Finally, we were able to leverage high-quality epigenetic data that have been uniformly processed by the Roadmap and ENCODE epigenome consortia. By utilizing DHS available across a subset of the 127 Roadmap epigenomes, the resolution of predicted functional elements was greatly improved and thus aided in reducing background variation less likely to be related to CRC.

Although these data aided in honing in on likely functional regions, the ARE we investigated only cover a small portion of the genome. On average, each cell-type specific ARE covered 3% of the autosome, leaving 97% of the genome untested in this analysis. In addition, the rare variants in this analysis were mainly imputed from a panel of 919 samples with 6x coverage WGS as opposed to more expensive sequencing at higher depths. While this internal sequencing panel has the advantage of being potentially enriched for risk variants given the higher proportion of CRC cases, lower coverage sequencing has decreased ability to confidently call rarer variants. Consequentially, we were unable to impute and test the CRC association for many of the true rare variants present in our study population. Based on the QC assessment we are confident in the calling of the variants from the WGS panel that were included in this study, and in the subsequent imputation. However, it should be noted that assessing the imputation quality of rarer variants remains an active area of study. Balancing sample size with sequencing coverage is an important consideration for rare variant studies. In light of the decreasing cost of WGS, we would recommend future studies to use higher coverage sequencing in order to better assess the burden of rarer regulatory variation. Another important consideration is the role of other types of functional elements both within the coding region (such as frameshift or splice variants) and in the non-coding regions (such as silencers, long non-coding RNA, and micro RNA). Furthermore, we had access to the epigenomes from only 3 non-diseased colorectal tissues. With more epigenetic data from relevant tissues and cell-types, the predicted ARE from CR and other digestive tissues would be more comprehensive and precise.

ARE defined by the Roadmap and ENCODE epigenetic data represent empirical estimates of the genomic location of enhancers and promoters. These elements regulate multiple genes and operate as nodes in gene expression networks. As is, we are treating the ARE as independent, distinct units. A logical next step is to define gene-enhancer links, where the entire regulatory-coding module can be treated as a unit of analysis (for each gene, define exons + promoter + enhancer). As new gene expression and epigenetic data become available, we will be able to define gene-enhancer links [22,23] and construct meaningful, statistically valid units of analysis for aggregate RVAM outside of the exome. In this study, we found that CR ARE were enriched for more significant CRC associations with both common and rare variants. Furthermore we found that this enrichment was not restricted to ARE of colorectal tissues. These findings may help guide novel approaches to discover biologically meaningful genetic associations for colorectal cancer and other complex diseases.

## Materials and methods

### Study participants and whole genome sequence imputed GWAS

#### Ethics statement

All participants gave written informed consent and this study has been approved by the Fred Hutchinson Cancer Research Center (FHCRC) Institutional Review Board.

#### GWAS participants

Study-specific eligibility criteria, details about genotyping, and quality control (QC) analyses can be found in Peters et al [24]. Briefly, we included individual-level genotype data pooled from a total of 12,661 CRC cases and 14,361 controls of European ancestry from 16 studies within the Genetics and Epidemiology of Colorectal Cancer Consortium (GECCO) and the Colorectal Cancer Family Registry (CCFR). Study characteristics are described in S5 Table. All cases were defined as invasive colorectal adenocarcinoma and confirmed by medical record, pathology report, or death certificate. Variants that were not directly genotyped on the GWAS platforms were imputed using an internal reference panel with a 2 to 1 CRC cases to control ratio with the intent of enriching for CRC risk variants.

#### Quality control of GWAS and WGS

610 CRC cases and 309 controls from the WHI study were sequenced with low-pass coverage and served as the internal WGS imputation panel for this study (S5 Table). Details about sequence data QC and imputation are described in Supporting Information text under Whole Genome Sequence Data and Genotype Imputation to GECCO-CCFR GWAS. In brief, multi-sample calling was performed at the University of Michigan. After removal of external duplicates, standard QC steps were used to examine for potential sample swap and contamination, e.g. sample heterozygosity, identity by descent (IBD) and principal component analyses. Only three samples were excluded from the analysis before imputation. For internal duplicates, the sample with the higher sequencing depth was selected for analysis. Gender checking confirmed all samples were female. Genotyping concordance between WGS and our previous GWAS were checked as well. Monomorphic, non-biallelic variants, and variants with low multi-sample calling quality (beagle R^2^<0.3) were removed from subsequent analysis. Distributions of sequencing depth, mapping quality, beagle allelic R^2^, minor allele frequency and minor allele count were examined for QC purposes.

### Definition of active regulatory elements, known loci, and rare variant sets

#### Description of active regulatory elements

Annotations for active regulatory elements (AREs) ARE were downloaded from Wouter Meuleman Reg2Map (http://www.broadinstitute.org/~meuleman/reg2map/HoneyBadger2_release/). Chromatin accessible regions were defined using the union of DNaseI-hypersensitivity sequencing (DNase-seq) peaks across 53 tissues and cell types. Enhancer and promoter states were previously annotated for the epigenomes of 127 tissues and cell-types (Roadmap + ENCODE) using a 5-mark 15-state Hidden Markov Model (ChromHMM) [25]. Active regulatory elements correspond to regions of chromatin accessibility within enhancer and promoter regions. DNase-seq signal scores of -log10(Poisson p-value) ≥ 10 were used as a threshold for statistically significant AREs because this was previously shown to provide very good separation between signal and noise [15].

We defined CR AREs using 2 rectal mucosa tissues (E101, E102) and one colon mucosa tissue (E075). We defined non-digestive AREs by excluding AREs from 12 digestive tissues (E075, E077, E079, E084, E085, E092, E094, E101, E102, E106, E109, E110), due to similarity with CR epigenomes [15]. Pairwise comparisons were made for ARE signal scores from each of the 127 tissues and cell types. Broad categorization of the 127 tissues into and cell-types was derived using hierarchically clustering using Pearson correlation as the distance measure and complete linkage followed by optimal ordering of leaves.

#### Defining rare variant sets

We anchored variant sets on transcription start sites (TSS), as defined by dbXref MySQL from UCSC Genome Browser (Human Feb. 2009 GRCh37/hg19 Assembly, data available at http://genome.ucsc.edu/cgi-bin/hgTables). For each set, we pooled variants within 200kb upstream and downstream of the TSS (S3 Fig). Only variant sets with a cumulative variance of ≥0.095 were analyzed, which is equivalent to analyzing single variants with MAF = 0.05. We calculated cumulative variance instead of cumulative MAF because variance can better account for genotype dosage imputation than MAF. In total, set-based CRC association tests were performed for 13,861 CR-ARE sets with 388,140 less frequent variants and 14,047 non-digestive ARE sets with 425,610 less-frequent variants. For CR tissue sets there was a median of 137 (interquartile range-IQR 91–197) variants per set. Similarly, for non-digestive sets there was a median of 94 (IQR 75–119) variants per set. The median cumulative variance across sets was 0.26 (IQR 0.17–0.38) and 0.17 (IQR 0.13–0.22) for CR sets non-digestive sets, respectively (S6 Table).

#### Defining known CRC loci

We defined the known colorectal cancer loci as 48 index variants likely to have independent effects on CRC risk and variants in LD with index variants (r^2^ ≥ 0.5; S3 Table). We calculated LD using Haploreg V3 [26] using EUR 1000 Genomes Phase 1 data and a 500kb maximum distance upstream and downstream for each LD region. Five of the loci were in LD (r^2^ ≥ 0.5) with a missense coding variant. Two of the five loci contained missense mutations (rs10936599, rs1789961) predicted by PolyPhen2 [27] (S3 Table) to have damaging effects. Given that none of the known loci have been confirmed through laboratory evidence to confer deleterious structural changes to the encoded protein, all 48 variants were included in the analysis. A polygenic risk score (PRS) was calculated as the sum of the imputed risk allele doses (ie the count of CRC risk increasing alleles carried).

### Statistical analyses

For variants with MAF ≥ 0.01 and imputation R^2^ ≥ 0.3, we ran marginal association analyses of the GWAS data using log-additive logistic regression of dosage effect on CRC risk with adjustment for age, sex (when appropriate), center (when appropriate), batch effects (ASTERISK only), and the first three PCs from EIGENSTRAT to account for population substructure within each individual study. To conduct functional set-based association analysis of rare variants, MAF < 0.01 and cumulative variance in set ≥ 0.095, we tested for association with CRC risk using MiST [19], adjusting for the same covariates as the single variant analysis. To test for enrichment, we compared the distribution of p-values for CRC association using the KS test. To test the hypotheses that, 1) ARE would be enriched for stronger CRC association p-values compared to non-ARE regions; and 2) ARE specific to CR would be enriched for stronger CRC association p-values compared to ARE specific to non-digestive cell-types, we tested enrichment using a one-sided KS test implemented using R version 3.2.2 (stats package function ‘ks.test’)[28].

Due to LD among variants, the asymptotic distribution for the KS test is not valid. We therefore used the resampling technique to estimate the p-value. Specifically, suppose there are X AREs in group 1 and Y AREs in group 2. A KS test was performed and the p-value was denoted as P_obs_. For each resampling, a random set of size X was drawn from the total X+Y AREs and assigned to group 1. The remaining AREs were assigned to group 2. The p-value from KS test was obtained, and denoted by P_sim_. The number of resamples varied from 10, 100, 1000, and up to 10,000 until 20% of the total number of p-values (P_sim_) were less than or equal to P_obs_. The empirical p-value was calculated as the ratio of the number of P_sim_ ≤ P_obs_ and the total number of resamples.

A p-value ≤ 0.05 was considered statistically significant. For 124 tissue comparisons the significance threshold was set to 0.05/124 and suggestive threshold was set to 0.05/19 given that several of the tissues examined were either biological duplicates (e.g. rectal mucosa samples from two donors, E101 and E102) or have highly similar epigenomes and cellular attributes (samples from the left versus right ventricles, E095 and E105).

For the comparison of CR versus non-digestive tissues the significance threshold was considered to be 0.05. For the 124 tissue comparisons the significance threshold was set to 0.05/124 and suggestive threshold was set to 0.05/19 given that many of the epigenomes were highly correlated.

## Supporting information

S1 Fig Relationships of ARE across 127 tissues and cell-types from Roadmap and ENCODE. AREs were defined as accessible chromatin regions (http://www.broadinstitute.org/~meuleman/reg2map/HoneyBadger2_release/DNase/p2/regions_all.bed, downloaded 2/9/2016) overlapping enhancer and promoter states marked by the H3K4me1 histone modification. Clustering was performed using the average H3K4me1 signal confidence scores, -log10(Poisson p-value), in the ARE of consolidated epigenomes for 127 tissues and cell-types (http://egg2.wustl.edu/roadmap/data/byFileType/signal/consolidated/macs2signal/pval/; downloaded 2/9/2016). The tissues and cell types were hierarchically clustered using Pearson correlation as the distance measure and complete linkage followed by optimal ordering of leaves. The leaves are colored by the broad categorization of each epigenome. Digestive tissues are colored in light purple. Immune cell-types are colored in green.
(PDF)

S2 Fig QQplot of CRC associations versus nondigestive tissue associations. The negative logarithm of the Colorectal ARE (y axis) and the combined Non-digestive ARE (x axis) CRC association P-value is plotted for each variant (dot). The red line indicates the null hypothesis that the two distributions of p-values are the same.
(TIF)

S3 Fig Detailed description of rare variant sets. UCSC genome browser image of an example variant set in the known *CDH1* locus. The first track shows the RefSeq gene annotations. The following 5 tracks are Roadmap ChIPseq histone modifications for colon mucosa. The following track ‘ChromHMM’ demarks the enhancer regions derived from the 15-state hidden markov modeling of these histone modifications. Next, the DHS marks across 52 Roadmap and ENCODE cell lines used to further refine likely transcription factors. The intersect of enhancer/promoter ChromHMM segments and DHS marks were used to define the Active Regulatory Elements (ARE) of colon mucosa. Variant sets were anchored on TSS as defined by protein coding gene transcripts with validated RefSeq records. If a gene had multiple TSS, the 5'-most and 3'-most TSS were used as anchors. Accordingly, variants overlapping ARE within 200Kb of a TSS were pooled into a test set, as shown in the highlighted blue region. Only sets with a cumulative variance of greater than 0.095 were analyzed.
(PDF)

S1 Table ARE enrichment of common variant CRC associations in comparison to non-ARE regions of the genome for 127 cell-types and tissues. Results for the kolmogorov-smirnov (KS) test for p-values of common variants in ARE vs those not in ARE are presented for 127 cell-types and tissues.
(XLS)

S2 Table Colorectal ARE enrichment of common variant CRC associations in comparison to non-colorectal ARE regions of the genome for 124 cell-types and tissues. Results for the kolmogorov-smirnov (KS) test for p-values of common variants in ARE of 3 CR tissues in comparison to ARE of another cell-type or tissues presented for 124 cell-types and tissues.
(XLS)

S3 Table Functional annotation of 48 known CRC loci. Functional annotation of 48 known loci defined as the Index variants and variants tagged by index with r^2^≥0.5 in all European 1000 Genomes Project populations (CEU+FIN+GBR+IBS+TSI).
(XLS)

S4 Table Single variant enrichment results from colorectal (CR) ARE versus 124 non-CR ARE using priority pruning at LD r^2^ thresholds of 0.9, 0.8 and 0.5. Results for the kolmogorov-smirnov (KS) test for p-values of common variants in CR ARE vs non-digestive tissue ARE across 124 cell-types and tissues.
(XLS)

S5 Table Descriptive characteristics of study population. (XLS)

S6 Table Rare variant set characteristics. (XLS)

S1 Text Supporting information. (DOCX)
