# Supplementary figures and images for: Enrichment of colorectal cancer associations in functional regions: Insight for using epigenomics data in the analysis of whole genome sequence-imputed GWAS data
Source: PLoS One. 2017 Nov 21;12(11):e0186518. doi: 10.1371/journal.pone.0186518 (PMC5697874; doi:10.1371/journal.pone.0186518)

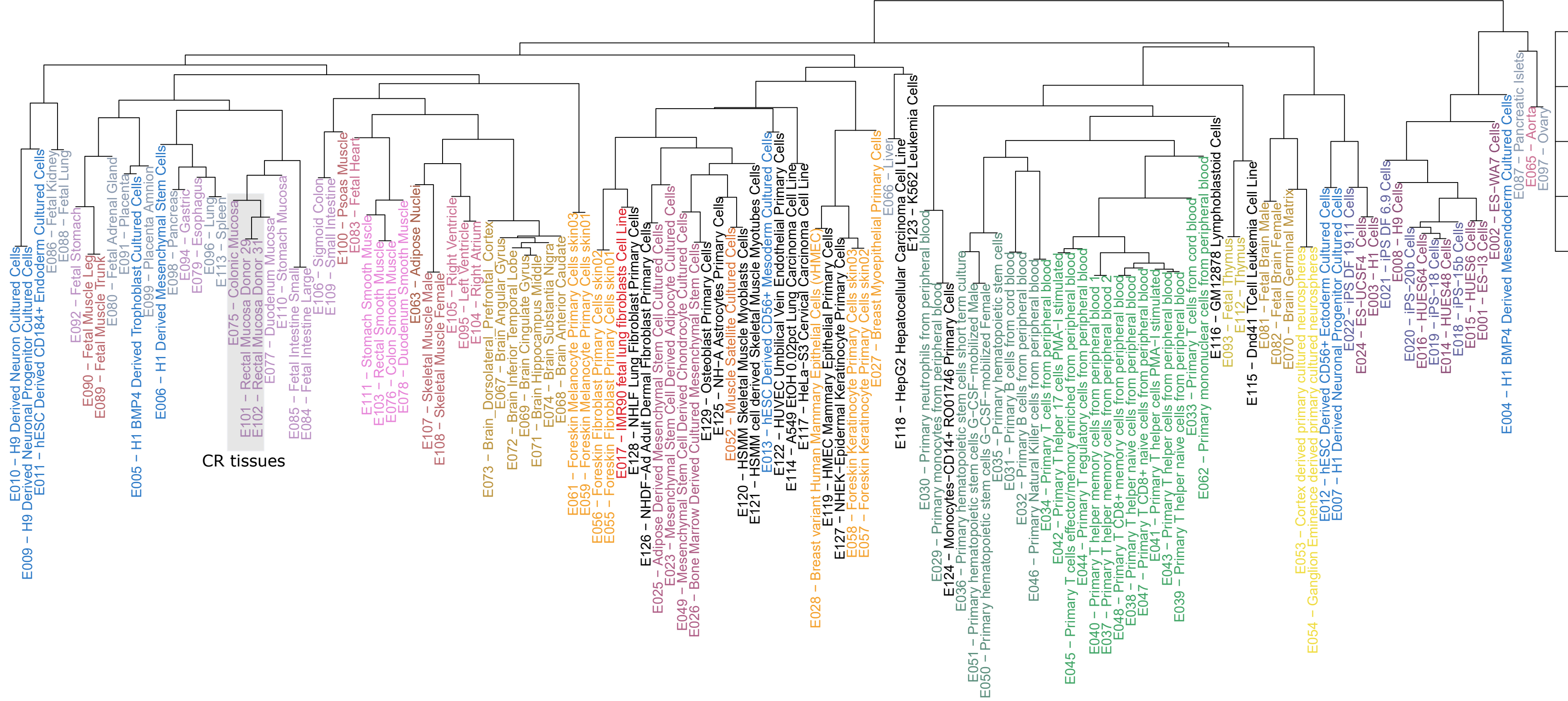

1 – Pearson correlation

Supplement: S1 Fig — AREs were defined as accessible chromatin regions (http://www.broadinstitute.org/~meuleman/reg2map/HoneyBadger2_release/DNase/p2/regions_all.bed, downloaded 2/9/2016) overlapping enhancer and promoter states marked by the H3K4me1 histone modification. Clustering was performed using the average H3K4me1 signal confidence scores, -log10(Poisson p-value), in the ARE of consolidated epigenomes for 127 tissues and cell-types (http://egg2.wustl.edu/roadmap/data/byFileType/signal/consolidated/macs2signal/pval/; downloaded 2/9/2016). The tissues and cell types were hierarchically clustered using Pearson correlation as the distance measure and complete linkage followed by optimal ordering of leaves. The leaves are colored by the broad categorization of each epigenome. Digestive tissues are colored in light purple. Immune cell-types are colored in green. (PDF) [file pone.0186518.s001.pdf]

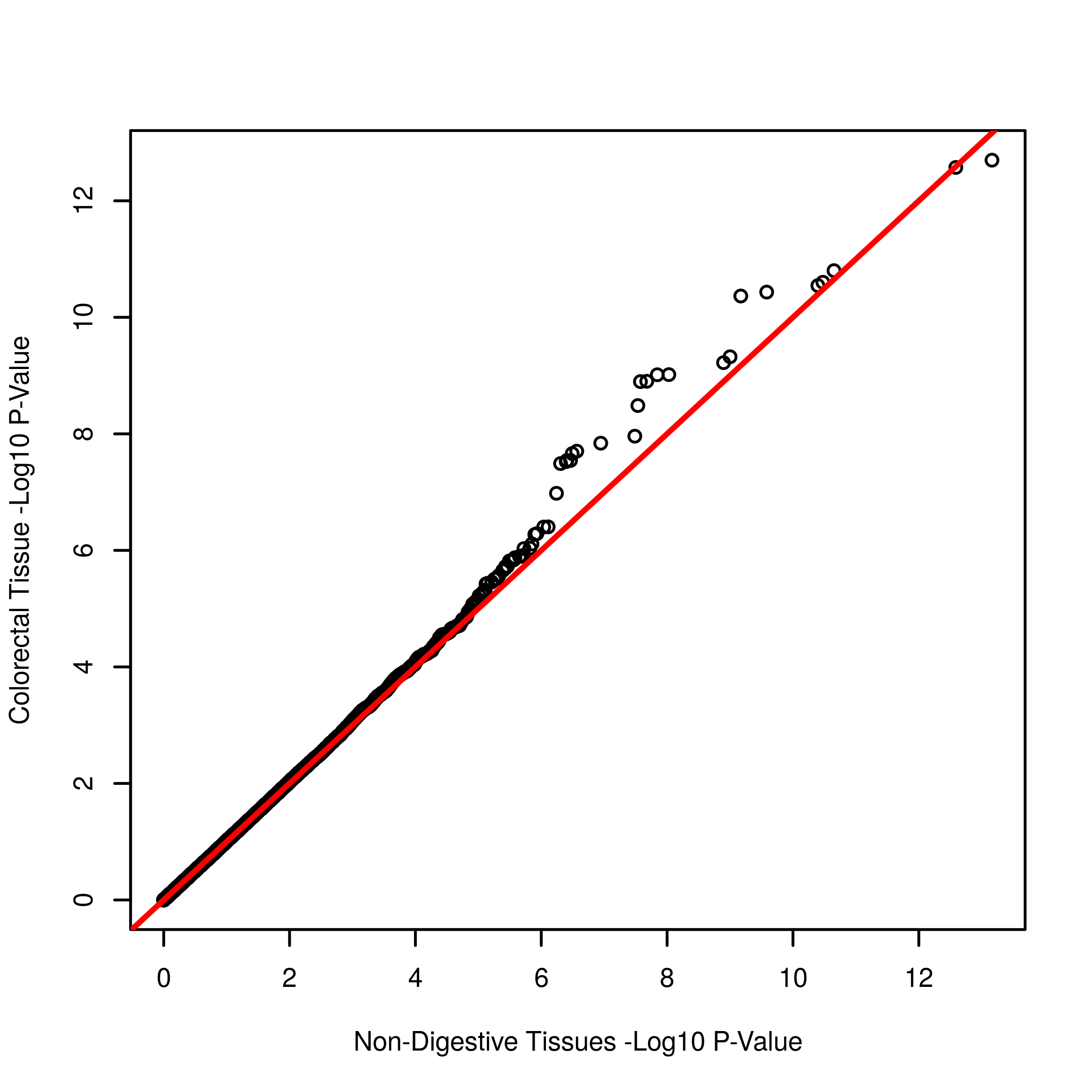

Supplement: S2 Fig — The negative logarithm of the Colorectal ARE (y axis) and the combined Non-digestive ARE (x axis) CRC association P-value is plotted for each variant (dot). The red line indicates the null hypothesis that the two distributions of p-values are the same. (TIF) [file pone.0186518.s002.tif]

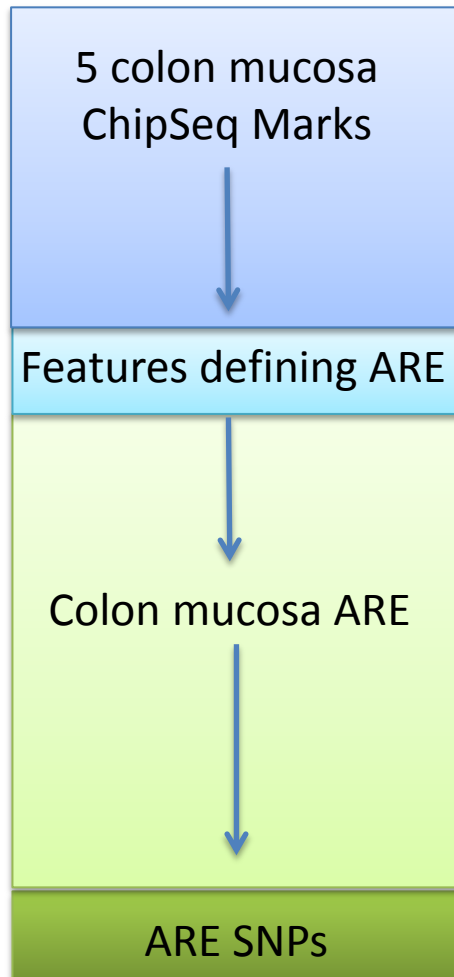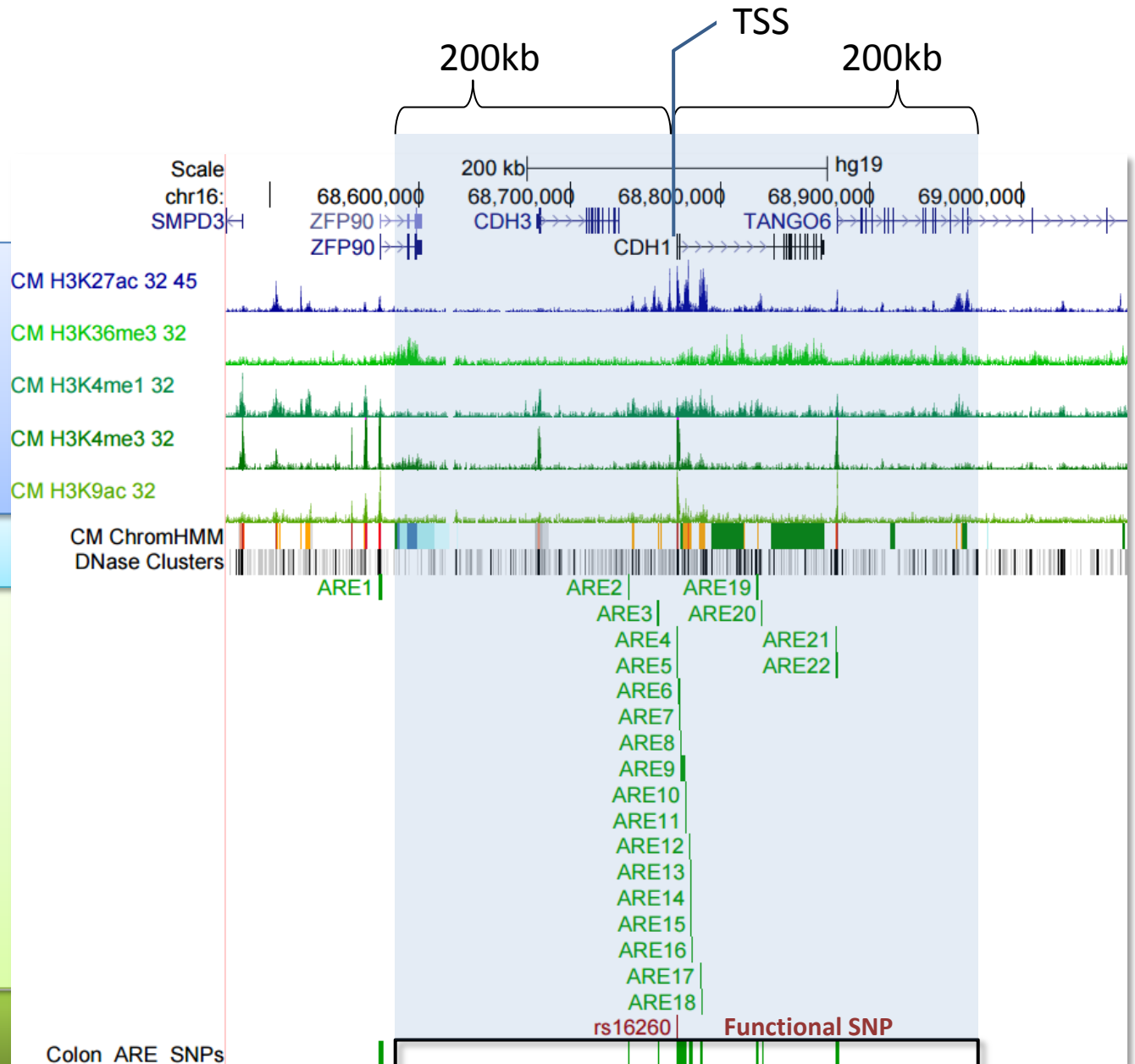

Functional Set Cumulative Variance > 0.095

Supplement: S3 Fig — UCSC genome browser image of an example variant set in the known CDH1 locus. The first track shows the RefSeq gene annotations. The following 5 tracks are Roadmap ChIPseq histone modifications for colon mucosa. The following track ‘ChromHMM’ demarks the enhancer regions derived from the 15-state hidden markov modeling of these histone modifications. Next, the DHS marks across 52 Roadmap and ENCODE cell lines used to further refine likely transcription factors. The intersect of enhancer/promoter ChromHMM segments and DHS marks were used to define the Active Regulatory Elements (ARE) of colon mucosa. Variant sets were anchored on TSS as defined by protein coding gene transcripts with validated RefSeq records. If a gene had multiple TSS, the 5'-most and 3'-most TSS were used as anchors. Accordingly, variants overlapping ARE within 200Kb of a TSS were pooled into a test set, as shown in the highlighted blue region. Only sets with a cumulative variance of greater than 0.095 were analyzed. (PDF) [file pone.0186518.s003.pdf]
