# Supplementary material for: Enrichment of colorectal cancer associations in functional regions: Insight for using epigenomics data in the analysis of whole genome sequence-imputed GWAS data
Source: PLoS One. 2017 Nov 21;12(11):e0186518. doi: 10.1371/journal.pone.0186518 (PMC5697874; doi:10.1371/journal.pone.0186518)
Supplement: S1 Text — (DOCX) [file pone.0186518.s010.docx]

**Supporting Information**

**Results**

**Methods**

**Whole genome sequence data**

We performed low coverage, whole-genome sequencing in 613 colorectal cancer (CRC) cases and 309 controls of European ancestry and 32 external controls from the Women’s Health Initiative[1,2] at the University of Washington (Seattle, WA, USA). Libraries were prepared with ThruPLEX-HD kits (Rubicon Genomics) and sequencing was performed using the Illumina HiSeq 2000 platform. Reads were mapped to the GRCh37 Human reference genome (<http://www.ncbi.nlm.nih.gov/projects/genome/assembly/grc/human/>) using the Burrows-Wheeler aligner (BWA, <http://bio-bwa.sourceforge.net/>) and stored as Binary Alignment Map (BAM) files. Genome Analysis Toolkit (GATK, http://broadinstitute.org/gatk) was used to remove systematic biases and to do quality recalibration. Genetic variants were called at the University of Michigan using the GotCloud variant calling pipeline[3] (genome.sph.umich.edu/wiki/GotCloud).

The average sequencing depth was 6.29. We filtered samples that exhibited relatedness based on identify by descent analysis (n=1), heterozygosity (n=0), sex mismatch (n=0), low concordance with genome-wide SNP array data (n=1), sample contamination (n=1), and genetic ancestry outliers as determined by principal component analysis (n=0). We explored potential batch effects. We removed monomorphic and non-biallelic variants (89,335), and variants with a calling Beagle r^2^ < 0.3 (2,785). The transition/transversion ration (Ti/Tv) genome-wide was 2.16. The Ti/Tv was very similar for variants with MAF <1% at 2.17. A total of 610 cases and 309 controls, and 21,256,065 autosomal variants remained after implementing this quality control (QC) pipeline.

**Genotype Imputation to GECCO-CCFR GWAS**

We pre-phased GWAS and sequencing data using BEAGLE [4] and imputed the WGS panel using Minimac [5]. Existing GWAS data from 24 colorectal cancer studies and 2 adenoma studies was imputed to the internal WGS panel (S5 Table) [6]. We used R^2^ as the imputation quality measure for imputed variants and applied R^2^ ≤ 0.3 filter for all common variants used in analyses. In total, we successfully imputed 21,256,065 autosomal markers before filtering on R^2^. After filtering on R^2^ there were 8,178,982 variants with a MAF ≥ 1% used in single common variant analyses. There were 12,993,665 variants with a MAF < 1% available for rare variant association analyses. For comparison, 10,080,522 of the less frequent/rare variants had an imputation R^2^ ≤ 0.3. However, rare variants were analyzed as aggregated sets. Therefore, a cumulative variance threshold for the set was used rather than imputation R^2^. Imputation quality was further investigated by comparing imputation R^2^ across three MAF bins for a subset of samples (n = 6,006) that were genotyped on the Illumina 730K array and imputed using both 1000 genomes phase 1 reference panel and our internal WGS panel (Fig A). Our internal WGS panel showed better imputation across the frequency spectrum. Box plots of the imputation R^2^ is shown for 5 different MAF bins in Fig B.


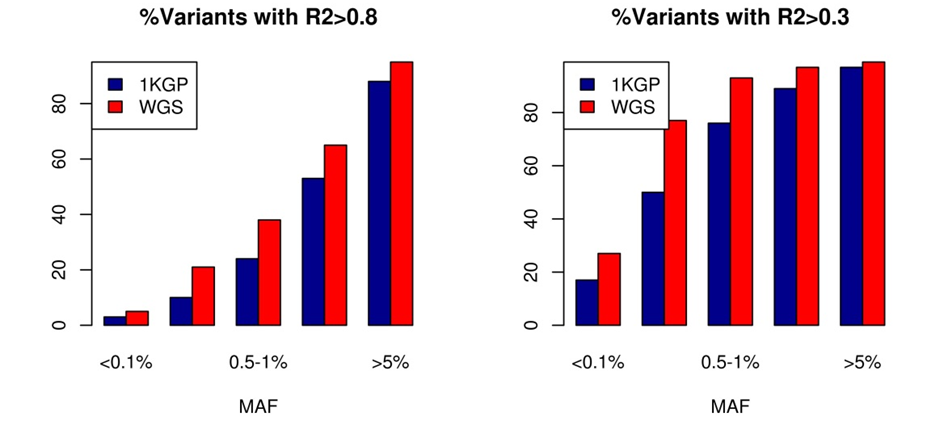


**Fig A. Comparison of imputation quality using One Thousand Genomes Project Phase 1 as a reference panel versus the internal WGS reference panel.** A subset of 6,006 samples were previously genotyped on the Illumina 730K array and used for imputation by both the thousand genomes project phase 1 (1KGP) panel (in blue) and our internal reference panel (in red) for comparison. Imputation quality was compared across five MAF bins. .


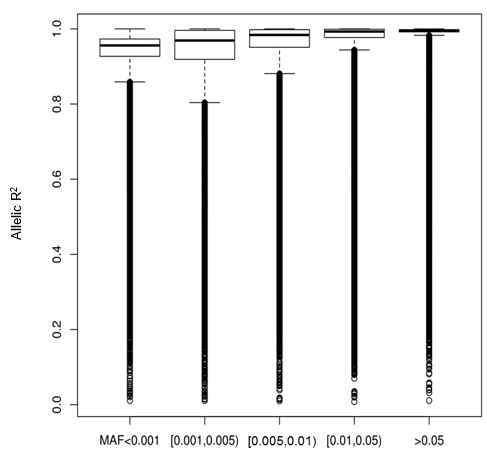


**Fig B.** **Allelic R^2^ from Beagle across minor allele frequency (MAF) bins.** Genotypes were re-called by Beagle based on LD information. The allelic R^2^ from Beagle is the squared correlation between the allele dosage with the highest posterior probability and the true allele dosage. It can be interpreted as the imputation quality for each variant. Variants with an R^2^ < 0.3 were removed.


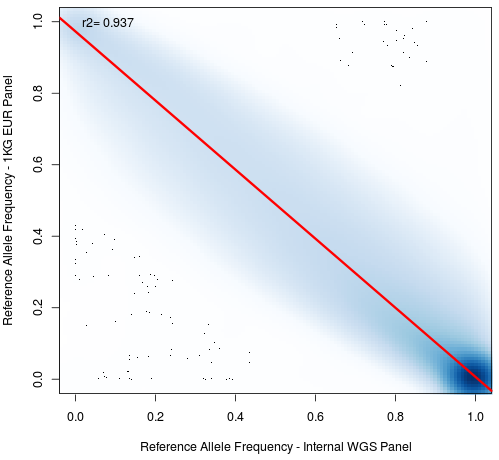


**Fig C.** **Comparison of reference allele frequency between EUR Thousand Genomes Phase 3 and imputed dosages from internal WGS reference panel.** Reference allele frequencies (GRCh37 human reference genome) for 14,834,157 variants present in both the Thousand Genomes Phase 3 reference panel and our imputed genotype data using an internal WGS reference panel were compared. Reference allele frequencies from the Thousand Genomes Phase 3 were taken from individuals of European ancestry (EUR). We observed a very strong Pearson’s correlation (r^2^ = 0.937).

**Description of Active Regulatory Elements**

Enrichment analyses were performed looking at active regulatory elements (AREs) across 127 tissues and cell-types. AREs represent chromatin accessible regions within enhancers and promoters. ARE data were downloaded from Wouter Meuleman Reg2Map (<http://www.broadinstitute.org/~meuleman/reg2map/HoneyBadger2_release/>). Chromatin accessible regions were delineated using the union of observed open chromatin DNaseI-hypersensitivity sequencing (DNase-seq) data across 53 tissues and cell types. Mapping of ARE has previously been described (1). In brief, enhancer and promoter states were annotated for the epigenomes of 127 tissues and cell-types (Roadmap + ENCODE) using a 5-mark 15-state Hidden Markov Model (ChromHMM) (1). Active regulatory elements correspond to the intersect between 1) open chromatin regions and 2) tissue and cell-type specific enhancer and promoter states that correspond to the H3K4me1 and H3K27ac histone modification marks. In order to identify statistically significant AREs, a signal confidence score was calculated as the Poisson p-value of DNase-seq counts relative to the expected dynamic background counts. DNase-seq counts at mappable genomic regions are calculated by counting the number of observed reads at that nucleotide position. The counts are then normalized to a dynamic Poisson background model in order to generate a per-base signal score. The -log10(Poisson p-value)≥ 10 was using as the threshold for significance because this was previously shown to provide very good separation between signal and noise (1).

Across the epigenomes of 127 tissues and cell-types, there were a total of 45,501 (1.24% of genome) putative promoter regions, 618,429 (7.46% of genome) putative enhancers, and 54,269 (0.75% of genome) putative regulatory regions that switch between the promoter and enhancer states (dyadic regions). Tissue-specific regulatory elements (promoter, enhancer and dyadic) were available for 3 colorectal mucosa tissues ― E101 rectal mucosa donor 29, E102 rectal mucosa donor 31, and E075 colonic mucosa. The union of all enhancer, promoter, and dyadic regions in these 3 tissues were defined as AREs in colorectal (CR) tissue. Additionally, tissue-specific regulatory elements were available for digestive cell-types, defined as: E075 colonic mucosa, E077 duodenum mucosa, E079 esophagus, E084 fetal large intestine, E085 fetal small intestine, E092 fetal stomach, E094 gastric, E101 rectal mucosa donor 29, E102 rectal mucosa donor 31, E106 sigmoid colon, E109 small intestine, E110 stomach mucosa.

**Comparison of AREs across Tissues and Cell-types**

We calculated Pearson correlation values between all pairwise combinations of reference epigenomes using the average per-base H3K4me1 signal confidence scores, -log10(Poisson p-value), within 200bp of an ARE. The resulting correlation matrices were used as the basis for a distance matrix for complete-linkage hierarchical clustering, followed by optimal leaf ordering(1). Results from this clustering are graphically displayed in Fig S1.

**Description of ARE Variant Annotation**

Using bedtools version 2.18 ([7], <http://bedtools.readthedocs.org/en/latest/>) we identified variants from the internal WGS-imputed GECCO-CCFR GWAS data that were positioned in the ARE for all 127 tissues and cell-types. After filtering on imputation r^2^ ≥ 0.3 and MAF ≥ 1% there was an average of 692,409 (range 376,795-1,111,338) ARE variants per epigenome. We observed an average of 109,489 (range 61,203-172,155) ‘rare’ ARE variants with 0.2% < MAF < 1%. The average mean of the imputation r^2^ for variants was 0.64 with an average minimum of 0.12 across epigenomes. For the set-based enrichment analyses, where variants were pooled within 200kb of a TSS, a cumulative variance threshold was set at 0.095. In other words, we obtained the variance of each genotype within a set and only kept sets whose cumulative genotype variance was greater than 0.095.

**Known CRC GWAS Loci and Derivation of Polygenic Risk Score**

The 48 known independent loci were defined as index SNPs with LD r^2^ ≤0.5 in 1000 genomes Phase 1 EUR and included in S3 Table. The genotype data described was used to construct polygenic risk scores (PRS) for the 48 known CRC loci. The PRS are calculated as the sum of the imputed risk allele doses (ie the count of CRC risk increasing alleles carried).

Where:

$N$ : Number of variants

$g_{ij}$ : Allelic dosage from imputation at variant i for individual j

**Statistical Analyses**

*CRC Association Tests*

Statistical analyses were conducted on pooled, individual-level data (S1 Table). To focus our analysis on only those variants and genes with sufficient power to detect an association, we conducted single variant analysis on variants with MAF greater than or equal to 1% and aggregate set-based testing among variants with MAF less than 1%. We used R version 3.2.2 to conduct all the statistical analyses.

*Single Variant-CRC Association Analysis*

We estimated the association between variants and risk for CRC using likelihood ratio tests with log-additive genetic effects. Each directly genotyped variant was coded as 0, 1, or 2 copies of the risk allele. For imputed SNPs, we used the expected number of copies of the risk allele (the “dosage”), which has been shown to give unbiased estimates in the association test for imputed SNPs [8]. We adjusted for age, sex, study, batch effects (ASTERISK only), and the first three principal components to account for population substructure. Inspection of the distribution of the p-values showed some evidence of over-dispersion of the test statistic, but was expected given inclusion of known CRC loci on the array. Calculation of the genomic inflation factor (λ) indicated little evidence of residual population substructure, cryptic relatedness, or differential genotyping between cases and controls (λ=1.063).

*Integrative Rare Variant Association Method Testing Set-CRC Association*

For the set-based testing we used the Mixed effect Score Test (MiST) method [9], which provides a general framework that includes a “burden” type test (such as the combined multivariate collapsing (CMC) [10]) as well as a variance components type test (such as the sequence kernel association test (SKAT)[11,12]. All analyses were adjusted for age, sex, study, batch effects (ASTERISK only), and the first three principal components.

**Supporting Information References**

1. Hays J, Hunt JR, Hubbell FA, Anderson GL, Limacher M, Allen C, Rossouw JE (2003) The Women's Health Initiative recruitment methods and results. Ann Epidemiol 13: S18-S77. S1047279703000425 [pii].

2. The Women's Health Initiative Study Group (1998) Design of the Women's Health Initiative clinical trial and observational study. Control Clin Trials 19: 61-109. S0197245697000780 [pii].

3. Jun G, Wing MK, Abecasis GR, Kang HM (2015) An efficient and scalable analysis framework for variant extraction and refinement from population-scale DNA sequence data. Genome Res 25: 918-925. gr.176552.114 [pii];10.1101/gr.176552.114 [doi].

4. Browning SR, Browning BL (2007) Rapid and accurate haplotype phasing and missing-data inference for whole-genome association studies by use of localized haplotype clustering. Am J Hum Genet 81: 1084-1097. S0002-9297(07)63882-8 [pii];10.1086/521987 [doi].

5. Li Y, Willer CJ, Ding J, Scheet P, Abecasis GR (2010) MaCH: using sequence and genotype data to estimate haplotypes and unobserved genotypes. Genet Epidemiol 34: 816-834. 10.1002/gepi.20533 [doi].

6. Peters U, Jiao S, Schumacher FR, Hutter CM, Aragaki AK, Baron JA, Berndt SI, Bezieau S, Brenner H, Butterbach K, Caan BJ, Campbell PT, Carlson CS, Casey G, Chan AT, Chang-Claude J, Chanock SJ, Chen LS, Coetzee GA, Coetzee SG, Conti DV, Curtis KR, Duggan D, Edwards T, Fuchs CS, Gallinger S, Giovannucci EL, Gogarten SM, Gruber SB, Haile RW, Harrison TA, Hayes RB, Henderson BE, Hoffmeister M, Hopper JL, Hudson TJ, Hunter DJ, Jackson RD, Jee SH, Jenkins MA, Jia WH, Kolonel LN, Kooperberg C, Kury S, Lacroix AZ, Laurie CC, Laurie CA, Le ML, Lemire M, Levine D, Lindor NM, Liu Y, Ma J, Makar KW, Matsuo K, Newcomb PA, Potter JD, Prentice RL, Qu C, Rohan T, Rosse SA, Schoen RE, Seminara D, Shrubsole M, Shu XO, Slattery ML, Taverna D, Thibodeau SN, Ulrich CM, White E, Xiang Y, Zanke BW, Zeng YX, Zhang B, Zheng W, Hsu L (2013) Identification of Genetic Susceptibility Loci for Colorectal Tumors in a Genome-Wide Meta-analysis. Gastroenterology 144: 799-807. S0016-5085(12)01846-X [pii];10.1053/j.gastro.2012.12.020 [doi].

7. Quinlan AR, Hall IM (2010) BEDTools: a flexible suite of utilities for comparing genomic features. Bioinformatics 26: 841-842. btq033 [pii];10.1093/bioinformatics/btq033 [doi].

8. Jiao S, Hsu L, Hutter CM, Peters U (2011) The use of imputed values in the meta-analysis of genome-wide association studies. Genet Epidemiol 35: 597-605. 10.1002/gepi.20608 [doi].

9. Sun J, Zheng Y, Hsu L (2013) A unified mixed-effects model for rare-variant association in sequencing studies. Genet Epidemiol 37: 334-344. 10.1002/gepi.21717 [doi].

10. Li B, Leal SM (2008) Methods for detecting associations with rare variants for common diseases: application to analysis of sequence data. Am J Hum Genet 83: 311-321. S0002-9297(08)00408-4 [pii];10.1016/j.ajhg.2008.06.024 [doi].

11. Lee S, Emond MJ, Bamshad MJ, Barnes KC, Rieder MJ, Nickerson DA, Christiani DC, Wurfel MM, Lin X (2012) Optimal unified approach for rare-variant association testing with application to small-sample case-control whole-exome sequencing studies. Am J Hum Genet 91: 224-237. S0002-9297(12)00316-3 [pii];10.1016/j.ajhg.2012.06.007 [doi].

12. Ionita-Laza I, Lee S, Makarov V, Buxbaum JD, Lin X (2013) Sequence kernel association tests for the combined effect of rare and common variants. Am J Hum Genet 92: 841-853. S0002-9297(13)00176-6 [pii];10.1016/j.ajhg.2013.04.015 [doi].
